# Development and Validation of Multiple Machine Learning Models Integrating Neutrophil‐Lymphocyte Ratio for Prediction of Hemorrhagic Transformation After Intravenous Thrombolysis in Acute Ischemic Stroke

**DOI:** 10.1111/cns.70667

**Published:** 2025-12-12

**Authors:** Fanhai Bu, Runlu Cai, Ying Hu, Xiaohong Tang, Wei Zhang, Xinxin Yang

**Affiliations:** ^1^ Department of Neurology, The First Clinical College Xuzhou Medical University Xuzhou Jiangsu China; ^2^ Department of Anesthesiology The First Affiliated Hospital of Xi'an Jiaotong University Xi'an China; ^3^ Department of Neurology Hongze District People's Hospital Huaian Jiangsu China; ^4^ Department of Neurology The Affiliated Hospital of Xuzhou Medical University Xuzhou Jiangsu China

**Keywords:** acute ischemic stroke, hemorrhagic transformation, intravenous thrombolysis, machine learning, prediction model, Shapley additive exPlanations

## Abstract

**Background:**

Hemorrhagic transformation (HT) is a critical complication of intravenous thrombolysis (IVT) in acute ischemic stroke (AIS). This study developed and validated machine learning (ML) models integrating inflammatory biomarkers with clinical indicators to predict post‐IVT HT.

**Methods:**

In 1272 IVT‐treated AIS patients, the least absolute shrinkage and selection operator (LASSO) regression identified five predictors from 17 variables, which were subsequently utilized to construct eight ML models. The models were trained (70% data) and tested (30% data). Furthermore, external validation conducted on an independent cohort substantiated the generalizability of the optimal model. The SHapley Additive exPlanations (SHAP) method explained feature importance.

**Results:**

LASSO screened five significant predictors: the neutrophil‐to‐lymphocyte ratio (NLR), admission National Institutes of Health Stroke Scale (NIHSS) score, the Alberta Stroke Program Early CT Score (ASPECTS), blood glucose, and atrial fibrillation. Logistic regression (LR) achieved optimal performance with an AUC of 0.833 internally and 0.842 externally. SHAP analysis prioritized NIHSS as the top contributor, while the nomogram elucidated the variability in HT risk.

**Conclusion:**

Integrating NLR with stroke severity and neuroimaging biomarkers enhances the accuracy of HT predictions. The LR‐based nomogram provided a practical tool for personalized IVT decisions, emphasizing the prognostic value of systemic inflammation in AIS management.

## Graphical abstract

Machine learning models integrating the inflammatory biomarker neutrophil‐lymphocyte ratio (NLR) with clinical/imaging biomarkers (NIHSS, ASPECTS) accurately predicted hemorrhagic transformation post‐thrombolysis. The optimal logistic regression‐based nomogram and SHAP analysis prioritized stroke severity and NLR for enhanced clinical utility.

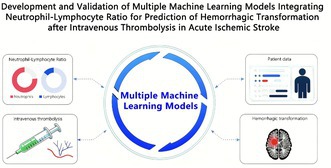

## 1 Introduction

Acute ischemic stroke (AIS) continues to be the second leading cause of mortality and long‐term disability globally, with intravenous thrombolysis (IVT) serving as the cornerstone of acute reperfusion therapy within established time windows [1, 2]. While IVT significantly improves functional outcomes (achieving modified Rankin Scale score ≤ 2 in 32%–42% of treated patients) [3], its clinical utility is substantially limited by hemorrhagic transformation (HT)—a spectrum of secondary hemorrhagic events occurring in 5.8%–7.3% of thrombolyzed cases, associated with a 3.9‐fold increase in mortality risk [4]. This complication arises from blood–brain barrier disruption due to ischemia–reperfusion injury and subsequent inflammatory cascades [5], underscoring the critical requirement for reliable predictive tools to optimize risk–benefit assessment.

Emerging evidence highlights systemic inflammation as a pivotal mediator in the pathogenesis of HT, with the neutrophil‐to‐lymphocyte ratio (NLR) being identified as a promising biomarker [6]. Nevertheless, the majority of current research primarily examines NLR through univariate frameworks, neglecting its synergistic interactions with clinical factors such as stroke severity and glycemic control [7]. This oversight limits clinical applicability, as isolated biomarkers rarely suffice for predicting complex outcomes.

In recent years, machine learning (ML) algorithms have shown transformative potential in developing predictive models for decoding complex biomedical patterns by deciphering multidimensional predictor‐outcome relationships [8, 9]. The least absolute shrinkage and selection operator (LASSO) regression, in particular, enables efficient feature selection from high‐dimensional datasets while mitigating multicollinearity—an essential advantage over conventional regression methods [10]. Applications of LASSO‐based ML methods have enhanced predictive accuracy in various medical fields [11, 12], including the prediction of the risk of HT after IVT [8, 13]. Therefore, combining NLR with other clinically relevant variables while harnessing a diverse array of ML classification models to develop a robust prediction model could potentially improve the predictive performance of HT [14].

To date, few studies have explored the incorporation of NLR into machine learning models for predicting HT following IVT, and the optimal approach has yet to be determined. In this study, we hypothesize that an NLR‐enhanced LASSO‐ML model will outperform current predictive tools by effectively capturing essential inflammatory–clinical interactions, thereby enabling precise identification of high‐risk HT patients and facilitating personalized treatment strategies.

## 2 Materials and Methods

### 2.1 Study Population

This retrospective cohort study was approved by the Ethics Committee of The Affiliated Hospital of Xuzhou Medical University (Approval number: XYFY2025‐KL044‐01). Written informed consent was obtained from all participants. The clinical data was collected from 1272 AIS patients who received IVT at our institution from September 2020 to July 2025. An independent external validation cohort consisted of AIS patients undergoing IVT at Hongze District People's Hospital between January 2019 and September 2023. Inclusion criteria for both cohorts were: (1) age > 18 years, (2) AIS diagnosed confirmed by clinical evaluation and neuroimaging examination, (3) onset of stroke symptoms within 4.5 h and treated with r‐tPA (0.9 mg/kg up to a maximum of 90 mg, 10% of the dose as a bolus followed by a 60‐min infusion of the remaining dose). Exclusion criteria included: (1) post‐IVT endovascular intervention, (2) missing data, (3) active malignancy, major trauma, rheumatic diseases, hematological disorders, or chronic inflammatory conditions, and (4) systemic infection within 2 weeks preceding admission.

### 2.2 Data Collection

The collected clinical variables encompassed: (1) demographic data: age and gender, (2) medical history: hypertension, diabetes mellitus (DM), coronary heart disease (CHD), atrial fibrillation (AF), anticoagulant therapy, antiplatelet therapy, smoking, and alcohol use, (3) clinical characteristics: systolic and diastolic blood pressure (SBP/DBP), onset‐to‐treatment time (OOT), National Institutes of Health Stroke Scale (NIHSS) score, Trial of ORG 10172 in Acute Stroke Treatment (TOAST) criteria, Alberta Stroke Program Early CT Score (ASPECTS) [15], (4) laboratory parameters: neutrophils, lymphocytes, platelets, eosinophils, blood glucose, albumin, glycated hemoglobin (HbA1c). The NLR was calculated as the ratio of absolute neutrophil count to lymphocyte count from admission complete blood tests.

### 2.3 Definition, Classification, and Subtype‐Specific Analysis of HT

AIS patients underwent baseline non‐contrast computed tomography (CT) within 4.5 h post‐onset. Subsequent evaluations using CT and magnetic resonance imaging (MRI) were performed to determine the occurrence of HT after IVT treatment, with additional imaging performed in cases of clinical deterioration [16, 17]. Two blinded neuroradiologists adjudicated HT subtypes based on the European Cooperative Acute Stroke Study III (ECASS III) [18]: Hemorrhagic Infarction (HI) and Parenchymal Hematoma (PH):small petechiae along the margins of the infarct (HI‐1) or confluent petechiae within the infarcted area but no space effect (HI‐2); blood clots in ≤ 30% of the infarcted area with some slight space‐occupying effect (PH‐1) or blood clots in > 30% of the infarcted area with substantial space‐occupying effect (PH‐2).

Due to the greater clinical impact of PH, a multi‐faceted comparative analysis was conducted: stratification into HI and PH subgroups, development of separate logistic models for each endpoint, AUC comparison between subtypes, and SHAP analysis to identify distinct feature importance patterns. This approach aimed to enhance early PH detection and provide pathophysiological insights.

Furthermore, stroke etiology was categorized using the TOAST criteria [19], and stroke severity was evaluated utilizing the NIHSS score at admission, post‐IVT, and discharge.

### 2.4 Feature Selection, Construction, and Evaluation of Predictive Models

Relevant factors were selected from independent variables, and patients were divided into training and test sets. Multiple ML classification models were employed to compare predictor significance and validate results. The best‐performing model was further evaluated, and SHAP was applied for global and individual interpretation.

#### 2.4.1 Feature Screening

LASSO regression (R, glmnet 4.1.8) was used for variable selection and regularization [10]. This regularization technique performed dual functions: (1) selecting HT‐associated predictors through L1‐penalization, and (2) developing a parsimonious predictive signature. Continuous variables were standardized (mean = 0, SD = 1). The optimal *λ* was determined through 10‐fold cross‐validation based on minimum criteria [9], selecting HT‐associated predictors while reducing overfitting.

#### 2.4.2 Data Division

The cohort was randomly split into 70% training and 30% testing sets using Python (v0.22.1) for model development and validation.

#### 2.4.3 ML Model Analysis

Eight ML models were utilized (Python: sklearn 0.22.1, xgboost 1.2.1, lightgbm 3.2.1) to develop the predictive model, including Logistic Regression (LR), Random Forest (RF), Extreme Gradient Boosting (XGBoost), Multilayer Perceptron (MLP), Support Vector Machine (SVM), Light Gradient Boosting Machine (LightGBM), Decision Tree (DT) and K‐Nearest Neighbors (KNN). LASSO‐selected features served as uniform inputs across all models. The logistic regression model was used to generate a Nomogram; other models underwent feature importance evaluation.

#### 2.4.4 Model Evaluation

Models were trained and evaluated through 10‐fold cross‐validation. Performance was assessed using: cutoff value, accuracy, precision, recall, F1 score, Receiver Operating Characteristic (ROC) curves with area under the curve (AUC) reported with 95% confidence intervals [20], calibration curves (Python, sklearn 0.22.1), and decision curve analysis (DCA; R, rmda 1.6) [21]. Generalizability was verified using learning curves [22] and external validation.

#### 2.4.5 SHAP Interpretation

SHAP (Python, shap 0.39.0) provided global feature importance, non‐linear relationship visualization, and individualized prediction explanations [23].

### 2.5 Statistical Analysis

Analyses were performed using R (v4.2.3), Python (v3.11.4), and SPSS (Statistical Package for the Social Sciences, v26.0). Continuous variable normality was evaluated with the Shapiro–Wilk test. Normally distributed data were presented as mean ± standard deviation (SD) and analyzed using the Independent Samples t‐test (for two‐group comparisons) or one‐way ANOVA (for multi‐group comparisons). Non‐normally distributed data were presented as median [interquartile range (IQR)] and analyzed using the Mann–Whitney *U* test (for two‐group comparisons) or the Kruskal–Wallis test (for multi‐group comparisons). Categorical variables were presented as frequencies (%) with associations assessed by the *χ* ^2^ test or Fisher's exact test, as appropriate. A two‐sided *p*‐value less than 0.05 was considered statistically significant.

## 3 Results

### 3.1 Baseline Characteristics of Patients With or Without HT

As shown in Figure S1, 1533 AIS patients receiving IVT were initially considered. After the exclusion of 106 patients due to post‐IVT endovascular intervention, 104 patients due to missing data, 30 patients due to active malignancy, major trauma, rheumatic diseases, hematological disorders, or chronic inflammatory conditions, and 21 patients due to systemic infection within 2 weeks preceding admission, the final analysis comprised 1272 patients. Baseline characteristics by HT status were presented in Table 1.

**TABLE 1:** Comparison of baseline characteristics between patients with or without HT.

Variables	Overall (*n* = 1272)	Non‐HT (*n* = 1098)	HT (*n* = 174)	*p*
*Demographics*				
Age, median (IQR)	69 (59, 77)	68 (59, 76)	74 (64, 80)	< 0.001
Gender (male, %)	824 (64.78)	723 (65.85)	101 (58.05)	0.055
*Vascular risk factors*				
Hypertension, *n* (%)	823 (64.70)	719 (65.48)	104 (59.77)	0.168
DM, *n* (%)	302 (23.74)	249 (22.68)	53 (30.46)	0.032
AF, *n* (%)	170 (13.37)	117 (10.66)	53 (30.46)	< 0.001
CHD, *n* (%)	220 (17.30)	172 (15.67)	48 (27.59)	< 0.001
Anticoagulant therapy, *n* (%)	101 (7.94)	74 (6.74)	27 (15.52)	< 0.001
Previous stroke, *n* (%)	384 (30.19)	330 (30.06)	54 (31.03)	0.863
History of smoking, *n* (%)	438 (34.43)	392 (35.70)	46 (26.44)	0.021
History of drinking, *n* (%)	221 (17.37)	196 (17.85)	25 (14.37)	0.308
*Clinical information*				
OTT, median (IQR)	184.5 (130, 237)	182 (127, 236)	201 (146, 240)	0.072
NIHSS on admission, median (IQR)	6 (4, 11)	6 (4, 9)	14 (8, 22)	< 0.001
NIHSS after IVT, median (IQR)	3 (2, 8)	3 (1, 6)	13.5 (6, 25)	< 0.001
NIHSS on discharge, median (IQR)	2 (0, 6)	2 (0, 4)	15 (4, 25)	< 0.001
Baseline SBP, median (IQR)	151 (137, 165)	150 (136, 165)	155.5 (140, 169)	0.027
Baseline DBP, median (IQR)	85 (77, 94)	85 (77, 94)	87 (77, 95)	0.650
ASPECTS, median (IQR)	8 (7, 8)	8 (7, 9)	7 (6, 7)	< 0.001
TOAST classification				
Large artery atherosclerosis, *n* (%)	849 (66.75)	734 (66.85)	115 (66.09)	< 0.001
Cardioembolism, *n* (%)	133 (10.46)	92 (8.38)	41 (23.56)	
Small vessel occlusion, *n* (%)	276 (21.70)	259 (23.59)	17 (9.77)	
Other determined and Undetermined, *n* (%)	14 (1.10)	13 (1.18)	1 (0.58)	
Hemorrhagic transformation, *n* (%)				
HI‐1	38 (2.99)		38 (21.84)	1.000
HI‐2	54 (4.25)		54 (31.03)	
PH‐1	46 (3.62)		46 (26.43)	
PH‐2	36 (2.83)		36 (20.69)	
*Laboratory signs*				
NLR, median (IQR)	2.97 (1.94, 5.06)	2.76 (1.88, 4.57)	4.81 (3.07, 7.40)	< 0.001
Platelets, median (IQR)	203 (166, 243)	204 (167, 243)	196 (160, 238)	0.404
Eosinophils, median (IQR)	0.08 (0.03, 0.15)	0.09 (0.04, 0.15)	0.05 (0.01, 0.12)	< 0.001
Blood glucose, median (IQR)	5.7 (4.9, 7.3)	5.5 (4.9, 6.9)	7.0 (5.7, 9.2)	< 0.001
Hemoglobin, median (IQR)	141 (132, 152)	141 (132, 152)	141 (129, 152)	0.608
CRP, median (IQR)	1.6 (0.6, 4.5)	1.5 (0.6, 4.3)	2.6 (0.9, 7.0)	< 0.001
Albumin, median (IQR)	42.3 (39.4, 45.0)	42.4 (39.7, 45.0)	40.9 (38.3, 44.6)	0.001
Triglyceride, median (IQR)	1.31 (0.97, 1.63)	1.32 (0.98, 1.65)	1.24 (0.92, 1.38)	0.003
LDL, median (IQR)	2.6 (2.1, 3.0)	2.6 (2.1, 3.0)	2.5 (2.0, 3.0)	0.439
HbA1c, median (IQR)	6.0 (5.6, 6.7)	6.0 (5.6, 6.6)	6.4 (5.7, 7.4)	< 0.001

Abbreviations: AF, Atrial fibrillation; ASPECTS, Alberta Stroke Program Early CT Score; CHD, coronary heart disease; DBP, diastolic blood pressure; DM, diabetes mellitus; HI, Hemorrhagic Infarction; HT, hemorrhagic transformation; IVT, intravenous thrombolysis; NIHSS, National Institutes of Health Stroke Scale; NLR, neutrophil‐to‐lymphocyte ratio; OTT, Onset‐to‐treatment; PH, Parenchymal Hematoma; SBP, systolic blood pressure.

The population had a mean age of 69 (range: 59–77), and 64.78% (*n* = 824) were male. Comparative analysis revealed significant disparities between HT (*n* = 174) and non‐HT (*n* = 1098) groups in multiple variables. Patients with NLR > 3.063 were more prone to hemorrhagic transformation (AUC = 0.696, odds ratio [OR] = 1.078, 95% CI = 1.037–1.120; *p* < 0.001). Compared to patients without HT, those in the HT group exhibited significant differences in several parameters, including age, blood glucose levels, admission NIHSS, ASPECTS, NLR, atrial fibrillation, coronary heart disease, anticoagulant therapy, diabetes, previous smoking, TOAST classification, SBP, HbA1c, eosinophils, albumin, CRP, and triglycerides (*p* < 0.05). No intergroup differences were observed in gender, hypertension, previous stroke, alcohol consumption, onset‐to‐treatment time, DBP, platelet count, LDL, and hemoglobin (all *p* > 0.05).

### 3.2 Comparison in Baseline Characteristics of the Training and Test Set

Patients were randomly allocated to a training set (*n* = 890) and a test set (*n* = 382) in a 7:3 ratio. No significant differences (*p* > 0.05) were observed in baseline characteristics between the sets (Table 2), confirming a balanced distribution with no systematic bias.

**TABLE 2:** Comparison of baseline data between the two datasets.

Variables	Training data (*n* = 890)	Test data (*n* = 382)	*p*
*Demographics*			
Age, median (IQR)	69 (60.78)	68 (58.76)	0.053
Gender (male, %)	578 (64.94)	246 (64.40)	0.902
*Vascular risk factors*			
Hypertension, *n* (%)	579 (65.06)	244 (63.87)	0.734
DM, *n* (%)	214 (24.05)	88 (23.04)	0.752
AF, *n* (%)	113 (12.70)	57 (14.92)	0.328
CHD, *n* (%)	150 (16.85)	70 (18.33)	0.579
Anticoagulant therapy, *n* (%)	70 (7.87)	31 (8.12)	0.970
Previous stroke, *n* (%)	268 (30.11)	116 (30.34)	0.981
History of smoking, *n* (%)	301 (33.82)	137 (35.86)	0.523
History of drinking, *n* (%)	153 (17.19)	68 (17.80)	0.855
*Clinical information*			
OTT, median (IQR)	182 (130, 236)	190 (130, 240)	0.354
NIHSS on admission, median (IQR)	6 (4, 11)	6 (4, 12)	0.367
NIHSS after IVT, median (IQR)	3 (2, 8)	3 (2, 9)	0.592
NIHSS on discharge, median (IQR)	2 (0, 6)	2 (0, 6)	0.912
Baseline SBP, median (IQR)	150 (137, 165)	152 (136, 166)	0.569
Baseline DBP, median (IQR)	85 (77, 94)	86 (77, 94)	0.840
ASPECTS, median (IQR)	8 (7, 8)	8 (7, 9)	0.585
TOAST classification			
Large artery atherosclerosis, *n* (%)	593 (66.63)	256 (67.02)	0.677
Cardioembolism, *n* (%)	89 (10.00)	44 (11.52)	
Small vessel occlusion, *n* (%)	199 (22.36)	77 (20.16)	
Other determined and Undetermined, *n* (%)	9 (1.01)	5 (1.31)	
*Laboratory signs*			
NLR, median (IQR)	3.05 (1.95, 4.84)	2.89 (1.91, 5.57)	0.785
Platelets, median (IQR)	202 (167, 244)	205 (164, 238)	0.947
Eosinophils, median (IQR)	0.08 (0.03, 0.15)	0.08 (0.03, 0.15)	0.843
Blood glucose, median (IQR)	5.6 (4.9, 7.2)	5.8 (5.0, 7.3)	0.465
Hemoglobin, median (IQR)	141 (129, 151)	141 (132, 152)	0.653
CRP, median (IQR)	1.7 (0.6, 4.6)	1.5 (0.5, 4.2)	0.215
Albumin, median (IQR)	42.3 (39.4, 44.9)	42.4 (39.4, 45.0)	0.952
Triglyceride, median (IQR)	1.3 (1.0, 1.6)	1.3 (0.9, 1.7)	0.751
LDL, median (IQR)	2.6 (2.1, 3.0)	2.6 (2.0, 3.0)	0.315
HbA1c, median (IQR)	6.0 (5.6, 6.6)	6.0 (5.6, 6.7)	0.698

Abbreviations: AF, Atrial fibrillation; ASPECTS, Alberta Stroke Program Early CT Score; CHD, coronary heart disease; DBP, diastolic blood pressure; DM, diabetes mellitus; HT, hemorrhagic transformation; IVT, intravenous thrombolysis; NIHSS, National Institutes of Health Stroke Scale; NLR, neutrophil‐to‐lymphocyte ratio; OTT, Onset‐to‐treatment; SBP, systolic blood pressure.

### 3.3 Feature Selection and Model Development

**FIGURE 1:**
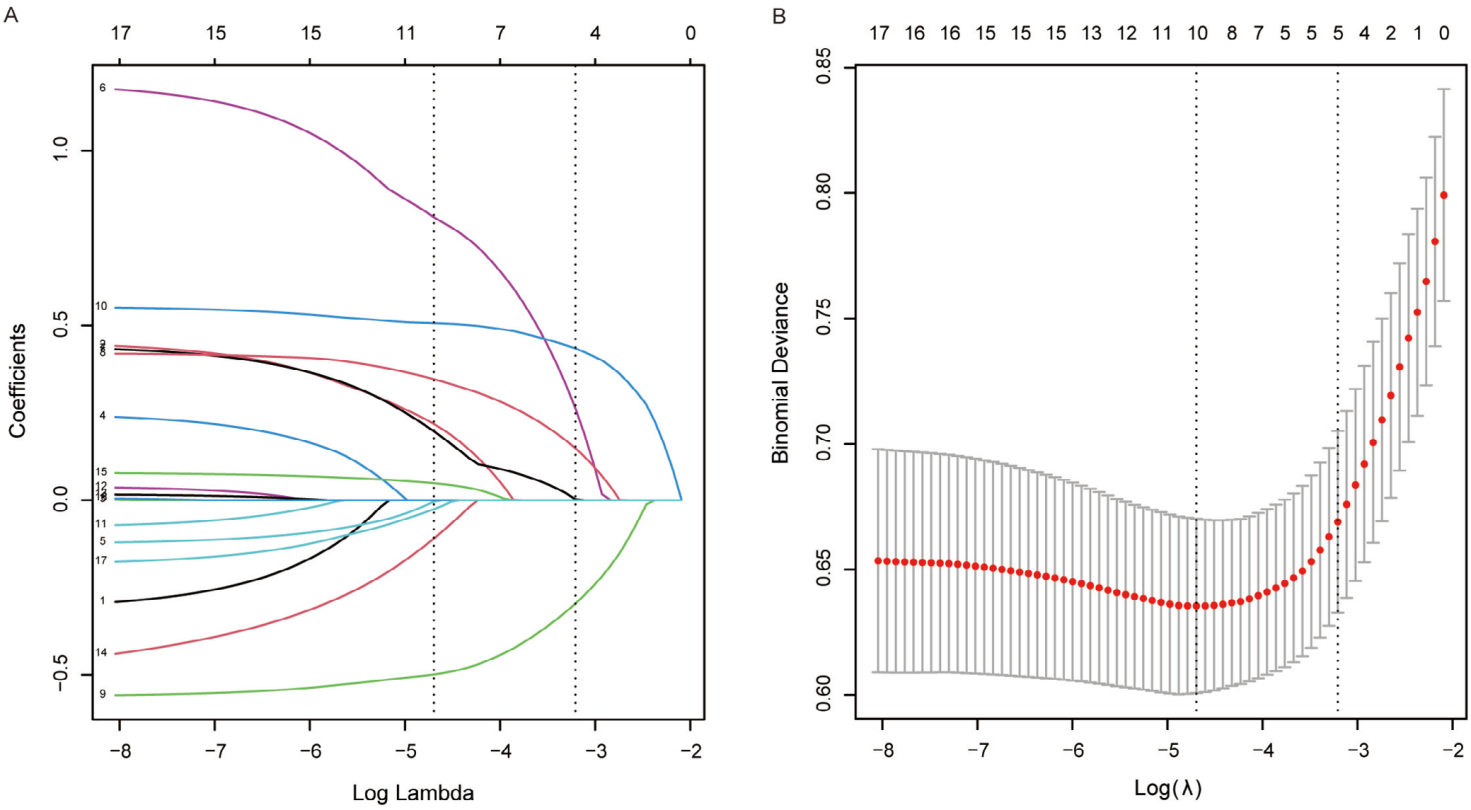
Feature selection based on LASSO regression. (A) Dynamic changes in regression coefficients: Visualizing the behavior of variable coefficients under L1‐regularized regression. (B) Optimization of the regularization parameter (*λ*): The ideal *λ* value was determined using 10‐fold cross‐validation to balance model complexity and predictive accuracy. Vertical dotted lines are drawn at the minimum mean square error (*λ* = 0.009) and the standard error of the minimum distance (*λ* = 0.04).

Univariate screening identified 17 variables with *p* < 0.05 (Table 1) for subsequent LASSO regression analysis in the training set with HT as the dependent variable. Using 10‐fold cross‐validation, LASSO (the standard error of the minimum distance *λ* = 0.04) selected five HT‐associated predictors from the initial 17 variables (Figure 1A). The selected features included atrial fibrillation, NLR, blood glucose, admission NIHSS score, and ASPECTS. Coefficient magnitudes are visualized in Figure 1B, demonstrating the directional associations of selected predictors. These five predictors were used in subsequent ML modeling. The LASSO coefficients are shown in Table S1.

### 3.4 Comparative Performance Evaluation of ML Models

Eight ML algorithms were implemented to predict post‐thrombolysis HT, including LR, RF, XGBoost, MLP, SVM, LightGBM, DT, and KNN. All models underwent 10‐fold cross‐validation to mitigate data partitioning bias, and learning curves for all models were analyzed to assess their generalization behavior. Model performance was compared using multiple indicators across training and testing sets.

**FIGURE 2:**
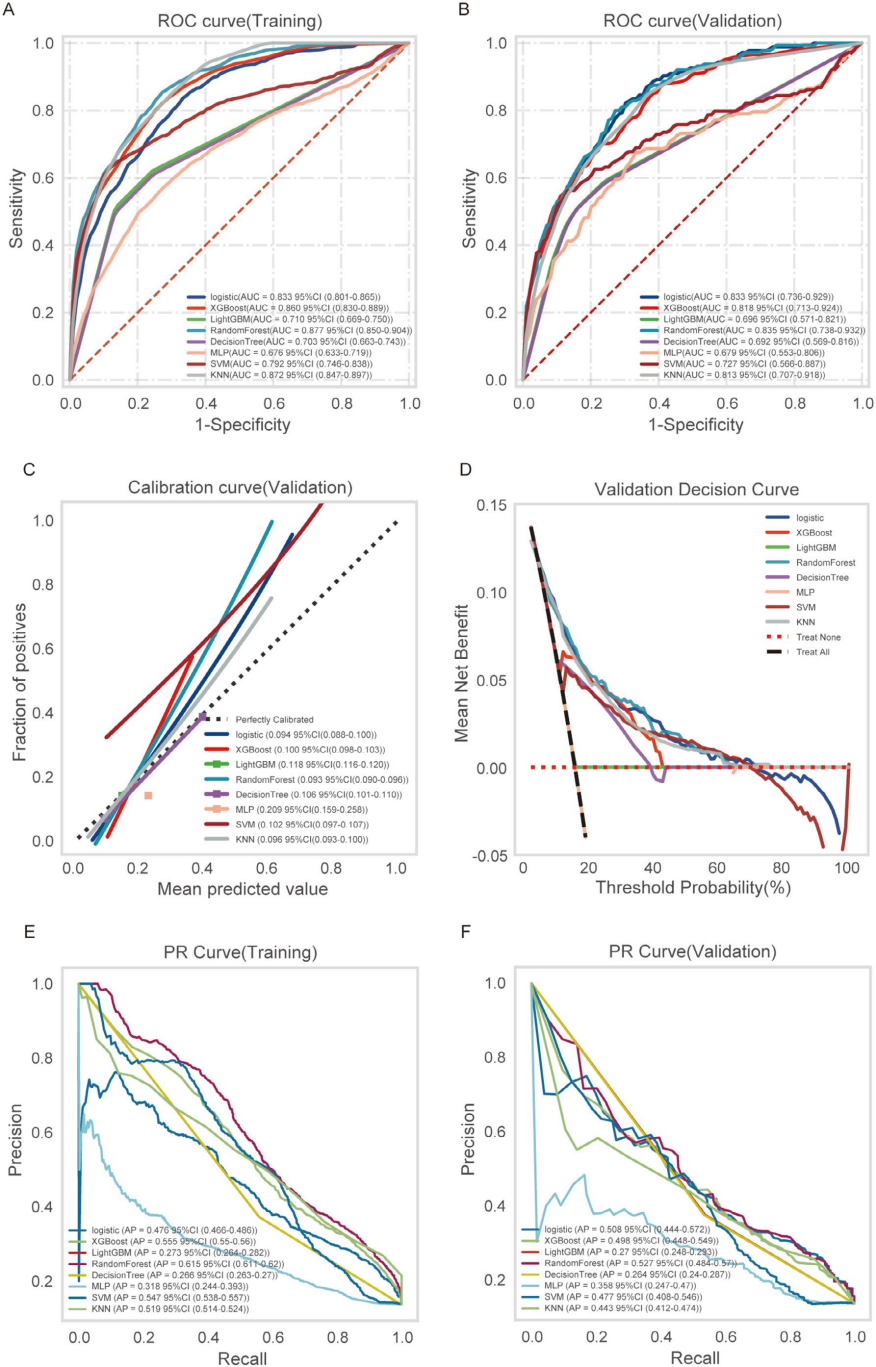
Comprehensive evaluation of machine learning models. (A) Receiver operating characteristic (ROC) curves and area under the curve (AUC) values for the training cohort. (B) ROC curves and AUC metrics for the testing cohort. AIS patient data were randomly partitioned 10 times using a 7:3 split ratio. (C) Calibration curves for the test set: The x‐axis displays the mean predicted probability, the y‐axis shows the observed event frequency, and the diagonal dashed line serves as the ideal reference. Smooth solid lines illustrate model‐specific calibration trends, with closer alignment to the reference line (and the smaller bracketed numerical values) indicating higher prediction accuracy. (D) Decision curve analysis (DCA) of the test set: The black dashed line corresponds to the clinical assumption that all patients experience hemorrhagic transformation (HT), while the red dashed line and thin black line reflect the scenario where no patients develop HT. Solid lines denote predictions from distinct ML models. (E) Training set PR curve and (F) testing set PR curve. Precision is plotted on the vertical axis, while recall is displayed on the horizontal axis. Superior model performance can be inferred when one model's PR curve entirely encompasses another's, with higher AP values indicating enhanced predictive capability. Distinct color schemes are utilized to differentiate between various models, with performance metrics expressed as mean values accompanied by 95% confidence intervals.

Based on ROC analysis [20], Logistic Regression demonstrated superior stability, achieving an identical AUC of 0.833 in both the training and validation datasets (Figure 2A,B). Its consistent performance was further confirmed by learning curves (Figure S2), and Delong's test results (Table S2). In contrast, Random Forest exhibited overfitting (∆AUC = −0.042; Figure 2B) with accuracy degradation from 0.759 to 0.729, XGBoost demonstrated unreliable probability calibration (Brier = 0.100 vs. 0.094 in LR; Figure 2C), while LightGBM showed fundamentally inadequate discriminative power (AUC = 0.696; Figure 2B; for more detailed insights, please refer to Table S3). Decision curve analysis affirmed the clinical utility of Logistic Regression across probability thresholds (Figure 2D). The model also achieved an optimal precision‐recall balance (AP = 0.508), outperforming tree‐based methods (Figure 2E,F). Given its computational efficiency, interpretability, and consistent performance, Logistic Regression was selected as the optimal model for clinical application.

### 3.5 Subtype‐Specific Predictive Analysis

Subtype‐specific analysis revealed substantial differences in predictive performance and feature importance between HI and PH pathogenesis. The logistic regression model showed superior discrimination for PH (AUC = 0.860, 95% CI: 0.820–0.900; Figure S3B) compared to HI (AUC = 0.760, 95% CI: 0.716–0.804; Figure S3A), underscoring its clinical value for detecting the more severe PH subtype. Both models utilized the same five predictors: atrial fibrillation, NLR, blood glucose, NIHSS score, and ASPECTS.

Feature importance analysis revealed distinct mechanistic drivers: HI prediction relied primarily on metabolic indicators (LDL) and platelet counts, suggesting microvascular endothelial leakage and impaired hemostasis (Figure S3C). PH prediction was dominated by neurological severity (NIHSS) and systemic inflammation (NLR), indicating core infarction processes with vascular rupture and neutrophil‐mediated damage (Figure S3D). These patterns affirm differing pathophysiology: HI is predominantly metabolic‐microvascular, whereas PH is neurological‐inflammatory. These insights support subtype‐specific monitoring—tracking NIHSS and inflammation for PH, and metabolic parameters for HI.

### 3.6 Binary Logistic Regression Analysis of HT Risk Factors and Nomogram Establishment

**FIGURE 3:**
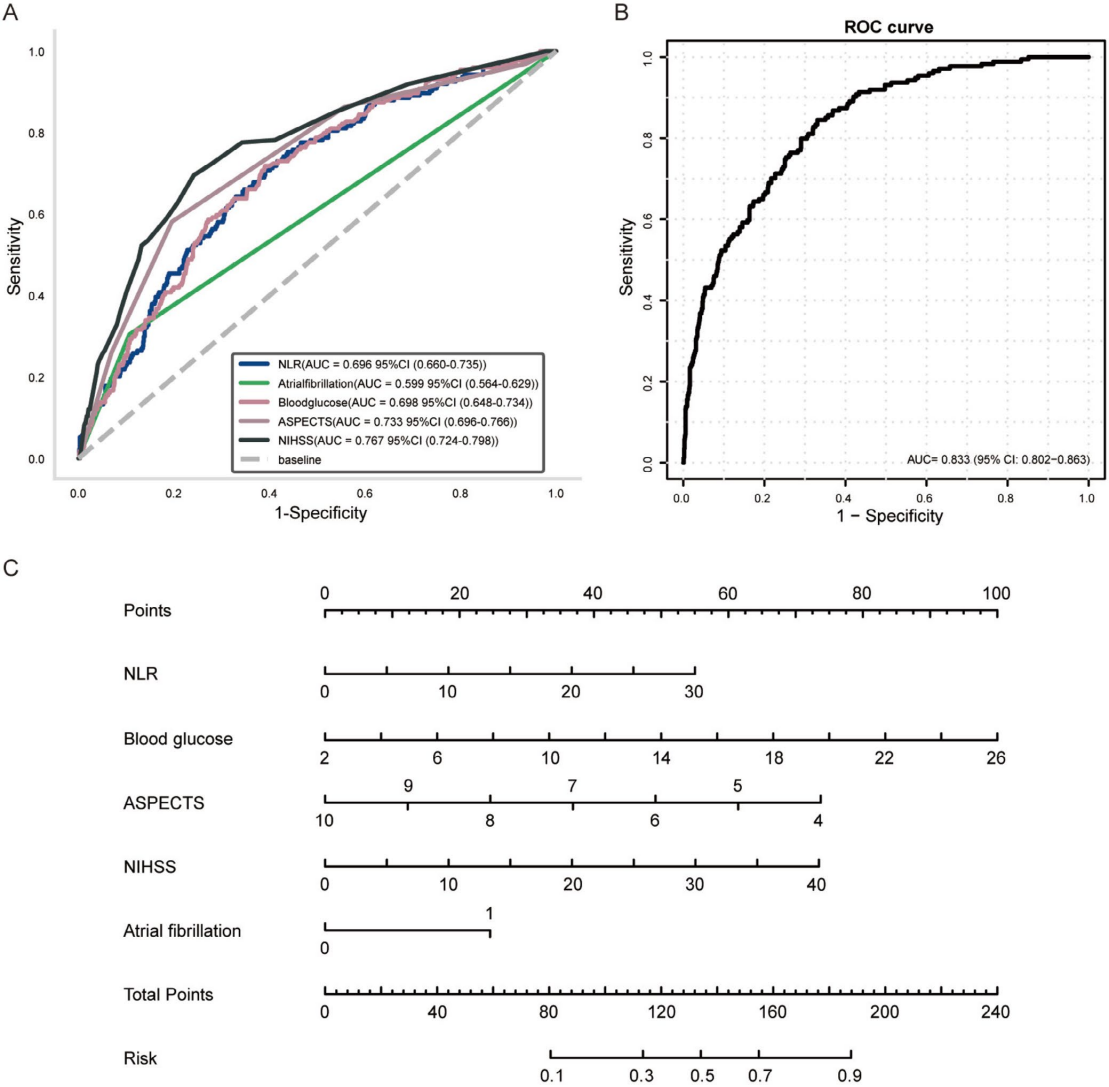
Area under the receiver operating characteristics curves (AUC) for predicting HT in AIS after Intravenous Alteplase. (A) Individual variables. (B) Combined variables. (C) Nomogram for HT risk prediction. Each predictor variable is assigned a point value based on its alignment with the upper scale. The summative score for an individual is calculated by aggregating the points assigned to all predictor variables.

LASSO‐selected variables were included in a multivariate Logistic Regression model (Table S4). After adjusting for confounders, NLR (OR = 1.078, 95% CI: 1.037–1.120, *p* < 0.001), NIHSS on admission (OR = 1.078, 95% CI: 1.055–1.101, *p* < 0.001), blood glucose (OR = 1.186, 95% CI: 1.117–1.259, *p* < 0.001), ASPECTS (OR = 0.604, 95% CI: 0.507–0.720, *p* < 0.001), atrial fibrillation (OR = 2.732, 95% CI: 1.767–4.226, *p* < 0.001) were identified as independent predictors of HT. ROC curves for individual variables and their combinations are shown in Figure 3A,B. Combining indicators yielded an AUC of 0.833, which was significantly higher than any single variable (Table S5).

Moreover, a nomogram was developed to quantify individual HT risk (Figure 3C), incorporating the five predictors: NLR (0–30 points), blood glucose (2–26 points), NIHSS (0–40 points), ASPECTS (4–10 points), and atrial fibrillation (0–1 point). The total score (0–240 points) corresponds to a predicted probability of HT (0–1.0), enabling rapid visual risk stratification.

### 3.7 Evaluation of Optimal Model Performance and Generalizability

**FIGURE 4:**
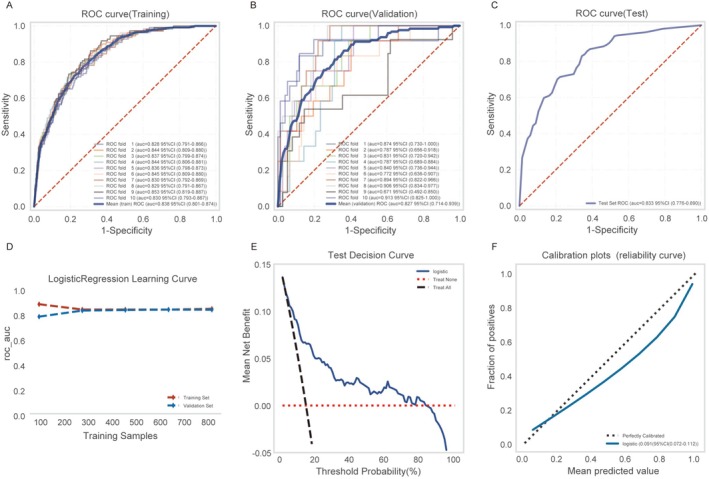
Development and evaluation of the Logistic Regression model involved three phases: Training, validation, and testing. (A) ROC curves and AUC values for the training cohort. (B) Validation cohort ROC analysis with AUC values. Model development utilized 10% of HT patient data for training and 10‐fold cross‐validation, where distinct colored solid lines depict outcomes from 10 iterations. (C) ROC performance and AUC results for the independent test cohort (30% of HT patients). (D) Learning curve progression, with red dashed and blue dashed lines indicating training and validation cohort performance, respectively. (E) Decision curve analysis (DCA) of the Logistic Regression model on the test set. (F) Calibration plot (reliability curve) of the logistic regression model. All metrics are reported as mean values with 95% confidence intervals.

The logistic regression model was rigorously evaluated using 10‐fold cross‐validation. Mean AUC values were 0.838 (95% CI: 0.801–0.874) for training subsets, 0.827 (95% CI: 0.714–0.939) for validation subsets, and 0.833 (95% CI: 0.776–0.890) for the test set (Figure 4A–C). All AUC values stabilized near 0.83, demonstrating consistent predictive accuracy with no overfitting (validation‐training ΔAUC = 0.011). This was further supported by the strong performance on the held‐out test set. The learning curve further confirmed strong generalization [22], with training and validation scores converging around 0.8 after 300 samples (Figure 4D). These findings collectively supported the applicability of the logistic regression model as an effective solution for the predictive modeling requirements of the given dataset.

Decision curve analysis showed decreasing net benefit with higher threshold probabilities, reflecting the model's clinical utility across thresholds (Figure 4E). Calibration plots indicated increasing observed event rates with higher predicted probabilities, signifying a higher proportion of correctly classified positive cases (Figure 4F). The integration of these analyses provided a comprehensive foundation for assessing model performance and its clinical utility.

### 3.8 SHAP Interpretation of the Predictive Model

**FIGURE 5:**
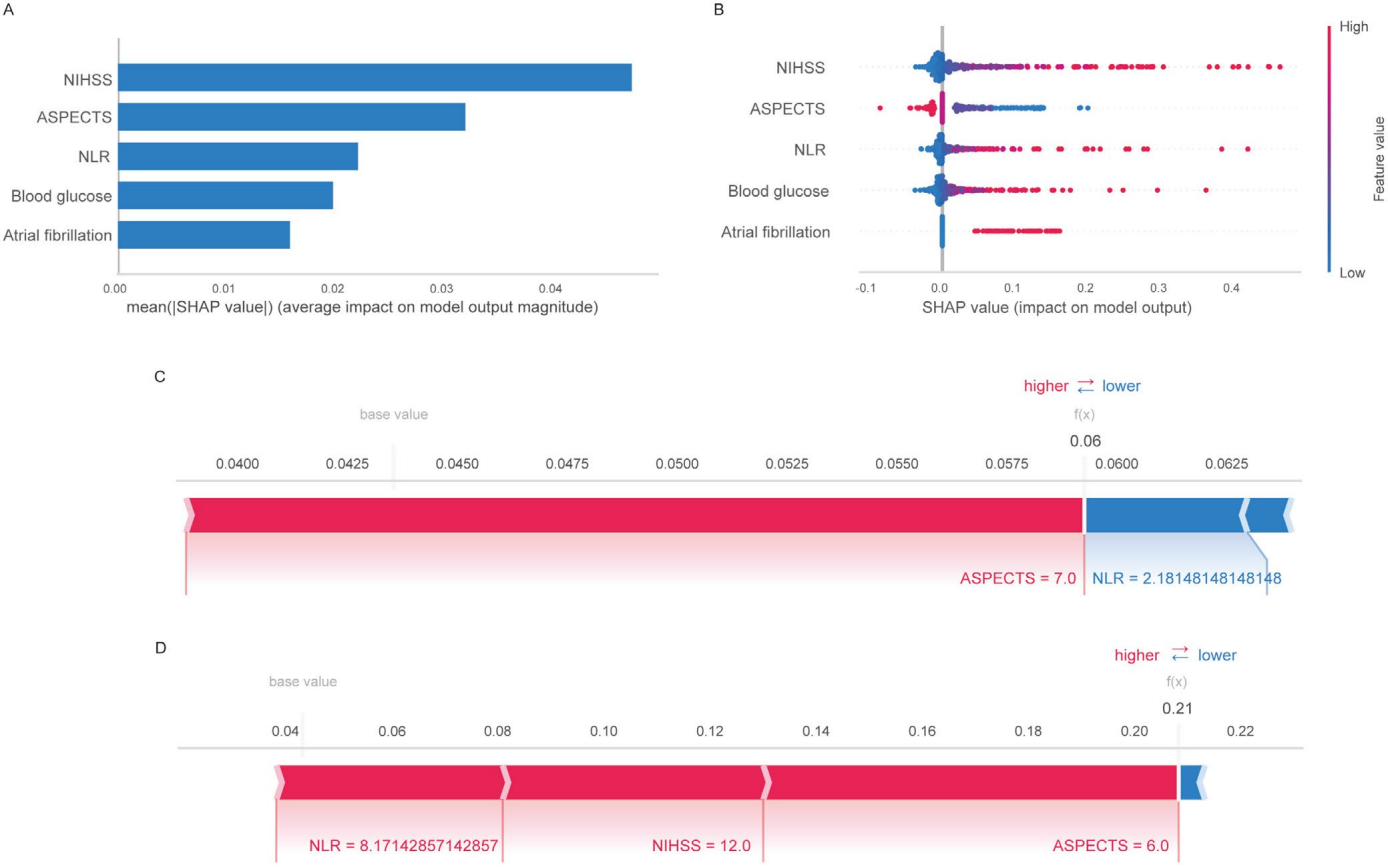
SHAP interpretation of the model. (A) SHAP‐based feature importance ranking: A matrix plot illustrates the relative contribution of each covariate to the final predictive model, prioritizing features by their impact magnitude. (B) Feature attributes visualized through SHAP values: Each horizontal line corresponds to a specific feature, with the *x*‐axis representing SHAP values. Red dots indicate higher feature values, while blue dots denote lower values. (C) SHAP force plot by AIS patients after IVT with non‐HT, and (D) with HT. SHAP values quantify feature‐specific contributions to model predictions, where higher absolute values indicate greater impact. Red markers denote above‐baseline feature values (risk‐enhancing), while blue markers represent below‐baseline values (risk‐reducing). Arrow lengths reflect effect magnitudes on log‐odds (*F*(*x*)), with longer arrows signifying stronger directional influences.

In recent years, ML models have gained traction in clinical decision support systems to predict outcomes and guide clinical decisions. But their black‐box nature creates interpretability barriers that hinder clinical adoption. To address this limitation in predicting the risk of post‐thrombolysis HT, we employed SHAP analysis for interpretability analysis [24, 25]. Global interpretability analysis quantified feature importance through mean absolute SHAP values, which represent each feature's average impact on the magnitude of the model output. As shown in Figure 5A, the NIHSS score demonstrated the strongest predictive influence, followed by the ASPECTS, NLR, blood glucose, and atrial fibrillation. The SHAP summary plot (Figure 5B) provides a more detailed view of feature contributions across the dataset, showing the five most important features. Each point represents an individual patient sample, with the color indicating the feature value (low to high). SHAP values provide a unified measure of feature importance and their impact on model predictions. Furthermore, two representative cases were analyzed to illustrate individual prediction explanations: a post‐IVT AIS case without HT exhibited diminished SHAP values (Figure 5C), contrasting with an HT‐positive counterpart displaying elevated predictive contributions (Figure 5D). This multi‐level SHAP interpretation demonstrates that interactions between biomarkers and clinical variables contribute to predictions, validating the model's pathophysiological rationality and providing clinically meaningful insights into feature contributions at both population and individual levels.

### 3.9 External Validation of the Logistic Regression Model

**FIGURE 6:**
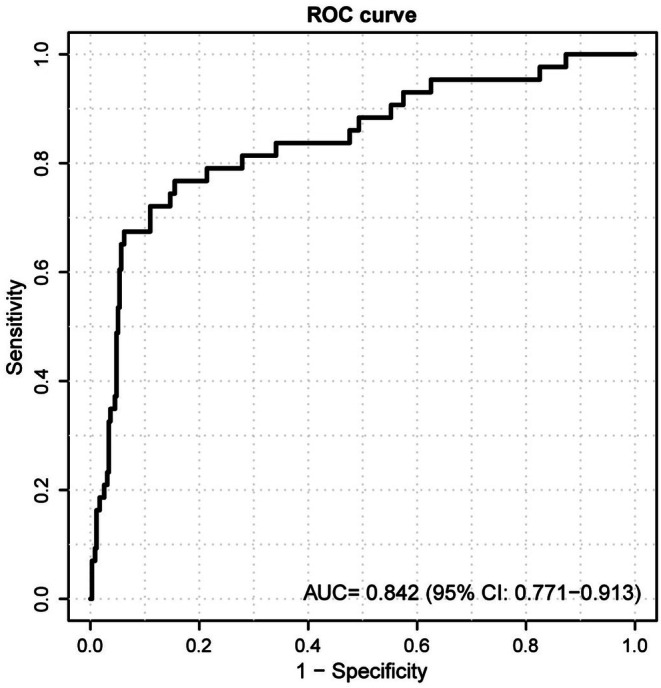
Area under the receiver operating characteristic curves (AUC) for AIS patients predicting HT after IVT in external datasets.

To further assess the generalizability, the established model underwent external validation on an independent cohort comprising 398 patients (HT = 43) sourced from another tertiary care center (Hongze District People's Hospital). The performance metrics demonstrated stable predictive accuracy with an AUC of 0.842 (0.771–0.913) (Figure 6), alongside a sensitivity of 0.842 and a specificity of 0.767. These results substantiate the robustness and external validity of the logistic regression predictive model across diverse clinical settings.

## 4 Discussion

The reported incidence of HT after IVT in AIS patients ranges from 10% to 48% [26, 27], with the cohort in this study demonstrating a 13.6% incidence rate aligning with prior reports. Predicting HT after IVT in AIS patients is crucial for optimizing treatment strategies and improving patient outcomes. HT is a significant complication that can lead to increased morbidity and mortality [1, 3, 5]. Accurate prediction of this complication is critical for therapeutic optimization and risk–benefit stratification. This study systematically integrated inflammatory biomarkers (NLR), neuroimaging parameters (ASPECTS), and clinical predictors (NIHSS, atrial fibrillation, blood glucose) through LASSO feature selection. The comparative analysis conducted on ML algorithms indicated that Logistic Regression demonstrated enhanced discriminative performance in comparison to other methods. The developed nomogram synthesizes these predictors into a bedside‐usable scoring system with three clinical advantages: Threshold guidance: 240‐point cutoff identifies high‐risk candidates for alternate reperfusion strategies; Dynamic monitoring: Serial NLR/glucose measurements enable real‐time risk recalibration; and Mechanistic transparency: Linear predictor interactions enhance clinician trust versus black‐box models.

### 4.1 Integrated Pathophysiological Framework for HT

The enhanced discriminatory capacity of our model stems from integrating inflammatory dysregulation with structural and clinical determinants. Notably, the integration of NLR into the model elevated predictive power beyond traditional risk factors, supporting the emerging hypotheses that systemic inflammation exacerbates blood–brain barrier (BBB) disruption after reperfusion [28, 29, 30]. NLR as inflammation–ischemia nexus: Neutrophils, elevated in acute stroke, release reactive oxygen species (ROS) and matrix metalloproteinases (MMPs), particularly MMP‐9, which degrade endothelial tight junctions and basal lamina [31]. Conversely, lymphopenia reflects the stress‐induced immunosuppression, impairing reparative mechanisms [32]. Crucially, neutrophils represent the earliest peripheral leukocytes recruited to cerebral tissue [33]. These cells mediate neurotoxic effects through multifaceted pathways including thrombus expansion, upregulated metalloproteinase activity, heightened production of oxygen free radicals, and neutrophil extracellular trap (NET) formation [34]. The resulting cascade, which includes increased capillary permeability, BBB compromise, and cellular edema, collectively impedes post‐stroke revascularization and vascular remodeling, thereby adversely compromising neural functional restoration. Robust clinical evidence confirms that early peripheral neutrophil elevation independently predicts neurological deterioration and unfavorable prognosis [35]. The NIHSS score and ASPECTS, widely recognized indicators of stroke severity and early ischemic changes, respectively, further strengthened the predictive power of our model. Higher NIHSS scores correlate with larger perfusion deficits and greater BBB permeability [36], while lower ASPECTS scores indicate more extensive early ischemic changes on computed tomography (CT) scans [37], highlighting the importance of early ischemic damage in HT development. These findings underscore the multifactorial nature of HT risk and by integrating these clinically relevant metrics, our model effectively captures the interplay between baseline stroke severity and structural brain damage in predicting HT outcomes [38]. Blood glucose, contributes to increasing free radical production and promoting platelet activation, further identified as a risk factor for HT [39]. Glycolysis produces cytotoxic acid products, aggravating vascular endothelial cell swelling, reducing microcirculation, and exacerbating vascular damage, further aggravating neurological deficits [40]. Additionally, atrial fibrillation (AF) drives HT through cardioembolic mechanisms where left atrial thrombi occlude cerebral arteries, causing ischemia–reperfusion injury that disrupts the neurovascular unit. This process increases blood–brain barrier permeability via MMP‐9‐mediated basal lamina degradation and microvascular fragility from embolic fragments [41]. The erratic cerebral blood flow patterns caused by atrial fibrillation lead to more unstable hemodynamics than sinus rhythm, triggering significant adverse cerebrovascular events [42]. Clinically, AF elevates HT risk 3.5‐fold post‐thrombolysis due to delayed reperfusion from slow emboli fragmentation and anticoagulant‐impaired clotting factor synthesis [43].

Our analysis revealed fundamental mechanistic divergences between HT subtypes: Parenchymal hematoma (PH) pathogenesis was driven by neuro‐inflammatory cascades (NIHSS/NLR dominance), where neutrophil‐mediated matrix degradation precipitates large‐vessel rupture. In contrast, hemorrhagic infarction (HI) involved metabolic‐microvascular disruption (LDL/platelet dynamics), characterized by endothelial leakage without structural failure. This dichotomy explains both PH's higher mortality risk and the AUC gap in detection performance (0.86 vs. 0.76 for HI). Crucially, NLR exhibited dual roles: direct vascular wall degradation in PH versus indirect endothelial repair impairment in HI. This multi‐hit model elucidates how inflammatory priming, structural vulnerability, and metabolic stress interactively drive HT pathogenesis [44].

### 4.2 Comparative Analysis of Machine Learning Models

While emerging studies have recently begun exploring ML for predicting HT post‐thrombolysis with demonstrated promising discriminative performance [45, 46], this study systematically evaluated and compared eight ML algorithms (Logistic Regression, Random Forest, XGBoost, MLP, SVM, LightGBM, DT and KNN) within a methodologically rigorous framework [47]. Stratified 10‐fold cross‐validation was employed to ensure algorithmic robustness and mitigate overfitting. The results revealed a notable performance‐interpretability tradeoff: while advanced ensemble methods such as Random Forest and XGBoost achieved high training AUCs (up to 0.877), they exhibited clinically substantial overfitting (∆AUC up to −0.042) and unreliable probability calibration. In contrast, logistic regression demonstrated unmatched stability with identical AUC (0.833) across training and validation sets, while maintaining clinically essential interpretability—making it the optimal choice for real‐world deployment. Moreover, independent external validation corroborated the result. This paradox highlights that model complexity does not inherently guarantee clinical translation potential, particularly when the inherent opacity of deep learning architectures hinders causal reasoning in medical decision‐making. To bridge this interpretability gap, the SHAP force plot aimed to further interpret the process of individualized HT risk prediction by the model, enabling us to comprehensively understand the prediction process and the contribution of features of the model to some extent.

### 4.3 Limitations

Although this study provided an effective and robust model in classifying the risk of post‐IVT HT, several limitations warrant acknowledgment. Firstly, the retrospective single‐center design risks unmeasured confounding. Additionally, limited statistical power from the modest sample size constrained granular evaluation of symptomatic intracranial hemorrhage (SICH) risk stratification. Lastly, manual neuroimaging interpretation introduces inter‐rater variability. Nevertheless, we still look forward to our model identifying patients who are at high risk for HT in a timelier manner.

## 5 Conclusion

This study establishes that a logistic regression model integrating atrial fibrillation, NIHSS score, ASPECTS, NLR, and blood glucose significantly enhances risk stratification accuracy for hemorrhagic transformation after intravenous thrombolysis in AIS patients. The developed clinically implementable nomogram provides quantitative risk estimation through visual weighting of these five predictors. In addition, we offer a personalized risk assessment for the development of post‐IVT HT in AIS patients as explained by SHAP. It may assist clinicians in making decisions regarding intravenous thrombolysis interventions for patients with AIS through an efficient computer‐aided approach.

## Author Contributions

Conceptualization: X.Y.; methodology: F.B. and R.C.; formal analysis: Y.H. and W.Z.; investigation: R.C. and Y.H.; data curation: X.T. and W.Z.; writing – original draft: F.B. and R.C.; writing – review and editing: X.Y.; visualization: W.Z.; supervision: X.Y. All authors critically reviewed the manuscript and approved the final version for publication.

## Ethics Statement

The studies involving human participants were reviewed and approved by the Research Ethics Committee of The Affiliated Hospital of Xuzhou Medical University (Approval number: XYFY2025‐KL044‐01). Written informed consent for participation was not required for this study in accordance with national legislation and institutional requirements.

## Conflicts of Interest

The authors declare no conflicts of interest.

## Supporting information


**Figure S1:** Flowchart of patient enrollment and exclusion criteria. AIS, acute ischemic stroke; HT, hemorrhage transformation; IVT, intravenous thrombolysis.
**Figure S2:** Learning Curves of Eight Machine Learning Models. Learning curves depict the model performance, evaluated by ROC AUC score (*y*‐axis), against the number of training samples (*x*‐axis). Each subplot corresponds to one of the following algorithms: (A) Logistic Regression, (B) Extreme Gradient Boosting (XGBoost), (C) Light Gradient Boosting Machine (LightGBM), (D) Random Forest (RF), (E) Decision Tree (DT), (F) Multilayer Perceptron (MLP), (G) Support Vector Machine (SVM), and (H) K‐Nearest Neighbors (KNN). In each panel, the training score is represented by the red diamond markers, and the validation score is shown with the blue diamond markers. The curves demonstrate the bias‐variance trade‐off for each model, with convergence behavior and generalization ability visually assessed through the gap and trend between training and validation scores.
**Figure S3:** Model performance and feature importance for hemorrhagic transformation prediction. (A) HI ROC curve; (B) PH ROC curve; (C) HI feature importance; (D) PH feature importance.

**Table S1:** The coefficients of the Lasso regression.
**Table S2:** The AUC DeLong test results.
**Table S3:** Predictive discriminative capacity across ML algorithms in the training and validation cohorts.
**Table S4:** Multivariate binary logistic regression for HT.
**Table S5:** Univariate analysis of key variables for HT.

## Data Availability

The data that support the findings of this study are available from the corresponding author upon reasonable request.
