# Supplementary figures and images for: Development and Validation of Multiple Machine Learning Models Integrating Neutrophil‐Lymphocyte Ratio for Prediction of Hemorrhagic Transformation After Intravenous Thrombolysis in Acute Ischemic Stroke
Source: CNS Neurosci Ther. 2025 Dec 12;31(12):e70667. doi: 10.1111/cns.70667 (PMC12700843; doi:10.1111/cns.70667)

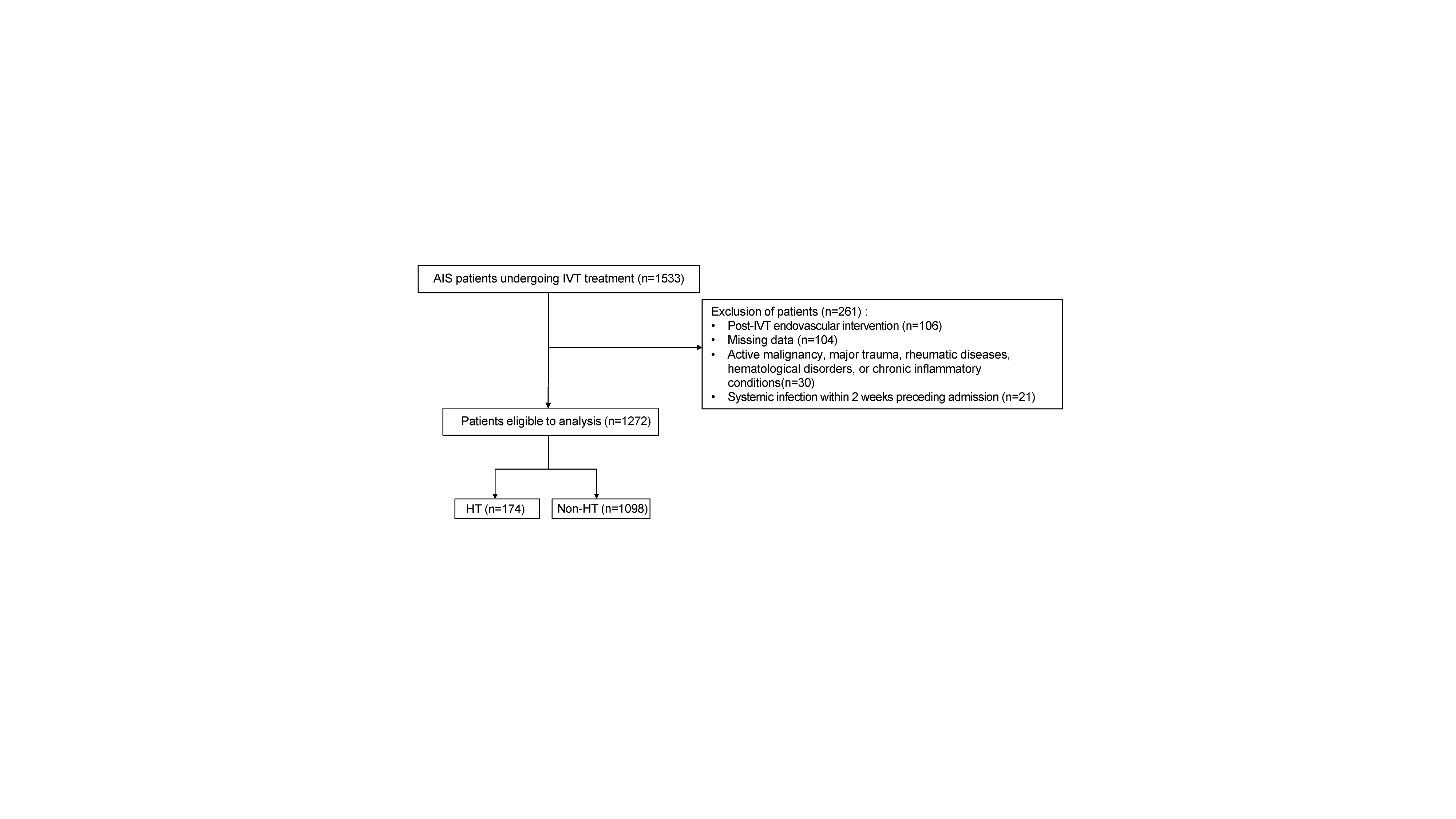

Supplement: Supplementary file 1 — Figure S1: Flowchart of patient enrollment and exclusion criteria. AIS, acute ischemic stroke; HT, hemorrhage transformation; IVT, intravenous thrombolysis. Figure S2: Learning Curves of Eight Machine Learning Models. Learning curves depict the model performance, evaluated by ROC AUC score (y‐axis), against the number of training samples (x‐axis). Each subplot corresponds to one of the following algorithms: (A) Logistic Regression, (B) Extreme Gradient Boosting (XGBoost), (C) Light Gradient Boosting Machine (LightGBM), (D) Random Forest (RF), (E) Decision Tree (DT), (F) Multilayer Perceptron (MLP), (G) Support Vector Machine (SVM), and (H) K‐Nearest Neighbors (KNN). In each panel, the training score is represented by the red diamond markers, and the validation score is shown with the blue diamond markers. The curves demonstrate the bias‐variance trade‐off for each model, with convergence behavior and generalization ability visually assessed through the gap and trend between training and validation scores. Figure S3: Model performance and feature importance for hemorrhagic transformation prediction. (A) HI ROC curve; (B) PH ROC curve; (C) HI feature importance; (D) PH feature importance. [file CNS-31-e70667-s001.zip › cns70667-sup-0001-FigureS1@Figure S1.tif]

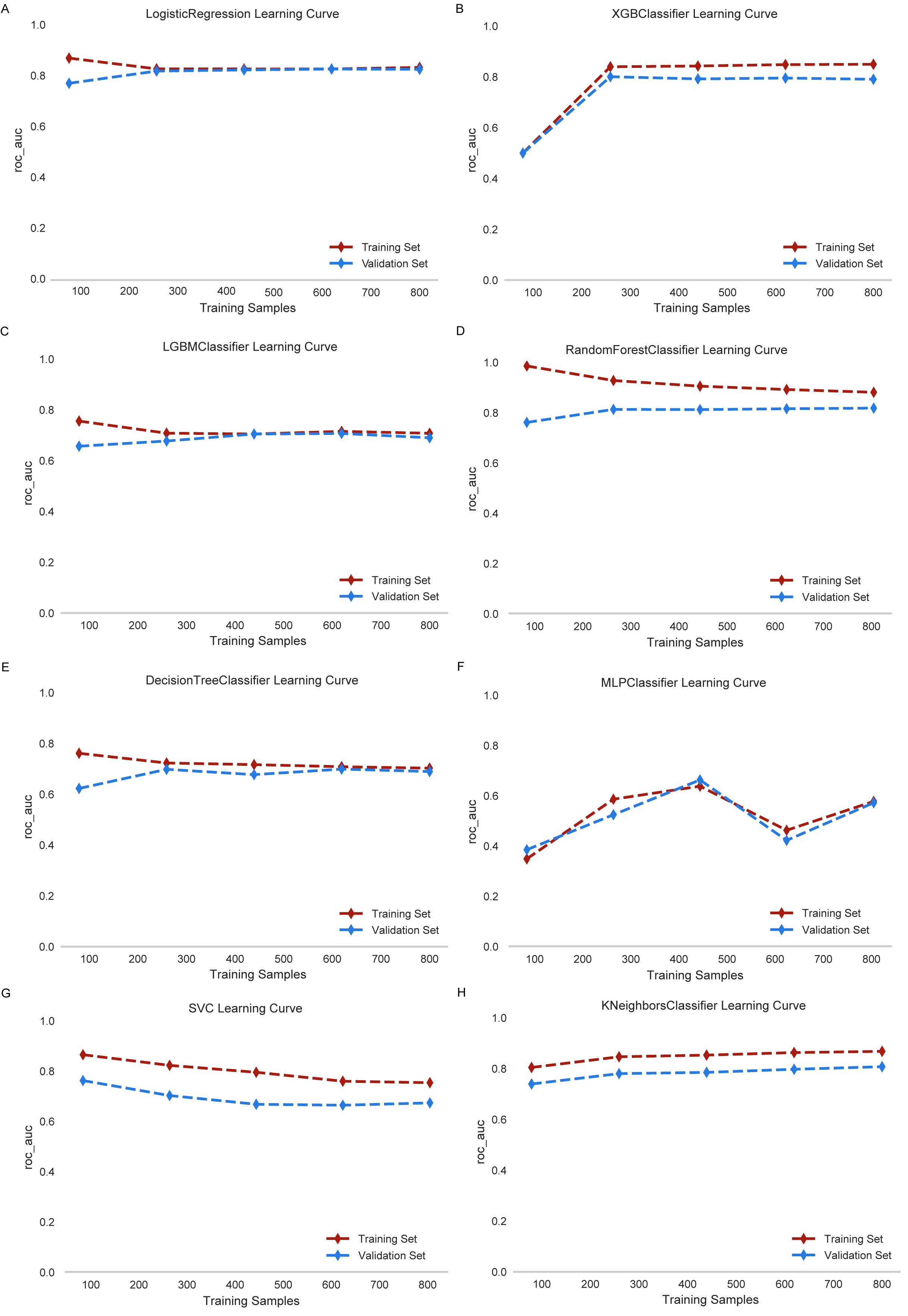

Supplement: Supplementary file 1 — Figure S1: Flowchart of patient enrollment and exclusion criteria. AIS, acute ischemic stroke; HT, hemorrhage transformation; IVT, intravenous thrombolysis. Figure S2: Learning Curves of Eight Machine Learning Models. Learning curves depict the model performance, evaluated by ROC AUC score (y‐axis), against the number of training samples (x‐axis). Each subplot corresponds to one of the following algorithms: (A) Logistic Regression, (B) Extreme Gradient Boosting (XGBoost), (C) Light Gradient Boosting Machine (LightGBM), (D) Random Forest (RF), (E) Decision Tree (DT), (F) Multilayer Perceptron (MLP), (G) Support Vector Machine (SVM), and (H) K‐Nearest Neighbors (KNN). In each panel, the training score is represented by the red diamond markers, and the validation score is shown with the blue diamond markers. The curves demonstrate the bias‐variance trade‐off for each model, with convergence behavior and generalization ability visually assessed through the gap and trend between training and validation scores. Figure S3: Model performance and feature importance for hemorrhagic transformation prediction. (A) HI ROC curve; (B) PH ROC curve; (C) HI feature importance; (D) PH feature importance. [file CNS-31-e70667-s001.zip › cns70667-sup-0003-FigureS2@Figure S2.tif]

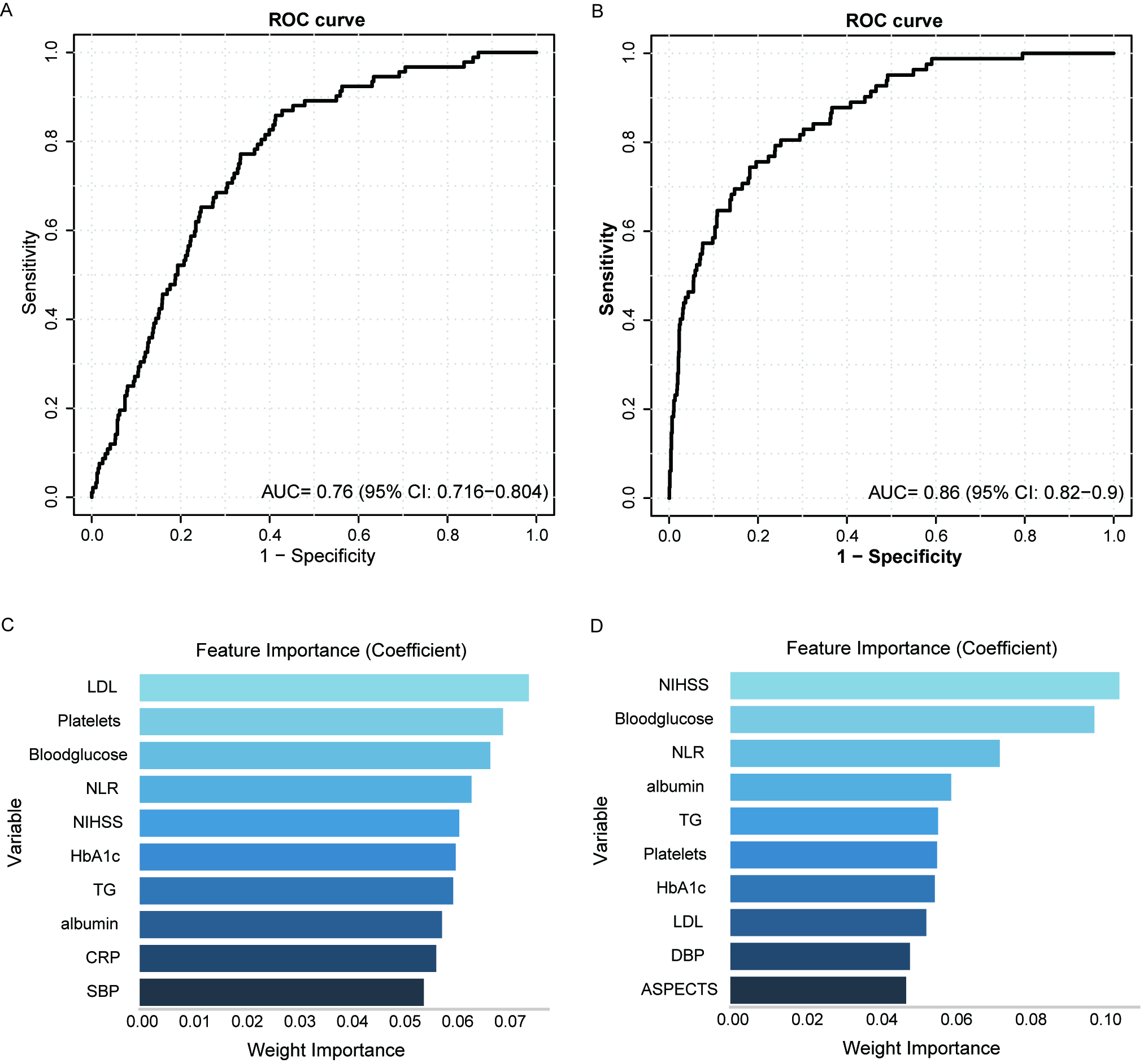

Supplement: Supplementary file 1 — Figure S1: Flowchart of patient enrollment and exclusion criteria. AIS, acute ischemic stroke; HT, hemorrhage transformation; IVT, intravenous thrombolysis. Figure S2: Learning Curves of Eight Machine Learning Models. Learning curves depict the model performance, evaluated by ROC AUC score (y‐axis), against the number of training samples (x‐axis). Each subplot corresponds to one of the following algorithms: (A) Logistic Regression, (B) Extreme Gradient Boosting (XGBoost), (C) Light Gradient Boosting Machine (LightGBM), (D) Random Forest (RF), (E) Decision Tree (DT), (F) Multilayer Perceptron (MLP), (G) Support Vector Machine (SVM), and (H) K‐Nearest Neighbors (KNN). In each panel, the training score is represented by the red diamond markers, and the validation score is shown with the blue diamond markers. The curves demonstrate the bias‐variance trade‐off for each model, with convergence behavior and generalization ability visually assessed through the gap and trend between training and validation scores. Figure S3: Model performance and feature importance for hemorrhagic transformation prediction. (A) HI ROC curve; (B) PH ROC curve; (C) HI feature importance; (D) PH feature importance. [file CNS-31-e70667-s001.zip › cns70667-sup-0004-FigureS3@Figure S3.tif]
