# Supplementary material for: Development and Validation of Multiple Machine Learning Models Integrating Neutrophil‐Lymphocyte Ratio for Prediction of Hemorrhagic Transformation After Intravenous Thrombolysis in Acute Ischemic Stroke
Source: CNS Neurosci Ther. 2025 Dec 12;31(12):e70667. doi: 10.1111/cns.70667 (PMC12700843; doi:10.1111/cns.70667)
Supplement: Supplementary file 2 — Table S1: The coefficients of the Lasso regression. Table S2: The AUC DeLong test results. Table S3: Predictive discriminative capacity across ML algorithms in the training and validation cohorts. Table S4: Multivariate binary logistic regression for HT. Table S5: Univariate analysis of key variables for HT. [file CNS-31-e70667-s002.docx]

**Table S1.** The coefficients of the Lasso regression

| **Variables** | **The minimum mean square error** | **The standard error of the minimum distance** |
| --- | --- | --- |
| Intercept | -3.417 | -2.301 |
| anticoagulant therapy | 0.0 | 0.0 |
| coronary heart disease | 0.218 | 0.0 |
| diabetes | 0.0 | 0.0 |
| previous smoke | 0.0 | 0.0 |
| TOAST classification | -0.004 | 0.0 |
| atrial fibrillation | 0.81 | 0.263 |
| NLR | 0.197 | 0.003 |
| blood glucose | 0.346 | 0.149 |
| ASPECTS | -0.499 | -0.296 |
| admission NIHSS | 0.506 | 0.434 |
| age | 0.0 | 0.0 |
| SBP | 0.0 | 0.0 |
| HbA1c | 0.0 | 0.0 |
| eosinophils | -0.11 | 0.0 |
| CRP | 0.048 | 0.0 |
| albumin | 0.0 | 0.0 |
| TG | -0.026 | 0.0 |

NLR, neutrophil-to-lymphocyte ratio; NIHSS, National Institutes of Health Stroke Scale; ASPECTS, Alberta Stroke Program Early CT Score; SBP, systolic blood pressure.

**Table S2.** The AUC DeLong test results.

| Model 1 | Model 2 | AUC 1 | AUC 2 | *P*-value |
| --- | --- | --- | --- | --- |
| MLP | LR | 0.676 | 0.833 | 0.255 |
| MLP | XGBoost | 0.676 | 0.860 | 0.320 |
| MLP | LightGBM | 0.676 | 0.710 | 0.217 |
| MLP | RF | 0.676 | 0.877 | 0.308 |
| MLP | DT | 0.676 | 0.703 | 0.222 |
| MLP | SVM | 0.676 | 0.792 | 0.427 |
| MLP | KNN | 0.676 | 0.872 | 0.375 |
| SVM | LR | 0.792 | 0.833 | 0.204 |
| SVM | XGBoost | 0.792 | 0.860 | 0.347 |
| SVM | LightGBM | 0.792 | 0.710 | 0.630 |
| SVM | RF | 0.792 | 0.877 | 0.201 |
| SVM | DT | 0.792 | 0.703 | 0.598 |
| SVM | KNN | 0.792 | 0.872 | 0.320 |
| RF | LR | 0.877 | 0.833 | 0.532 |
| RF | XGBoost | 0.877 | 0.860 | 0.308 |
| RF | LightGBM | 0.877 | 0.710 | 0.006 |
| RF | DT | 0.877 | 0.703 | 0.006 |
| RF | KNN | 0.877 | 0.872 | 0.438 |
| KNN | LR | 0.872 | 0.833 | 0.539 |
| KNN | XGBoost | 0.872 | 0.860 | 0.612 |
| KNN | LightGBM | 0.872 | 0.710 | 0.068 |
| KNN | DT | 0.872 | 0.703 | 0.065 |
| LightGBM | LR | 0.710 | 0.833 | 0.026 |
| LightGBM | XGBoost | 0.710 | 0.860 | 0.029 |
| XGBoost | LR | 0.860 | 0.833 | 0.507 |
| XGBoost | DT | 0.860 | 0.703 | 0.029 |
| LR | DT | 0.833 | 0.703 | 0.026 |

LR, Logistic Regression; RF, Random Forest; XGBoost, Extreme Gradient Boosting; MLP, Multilayer Perceptron; SVM, Support Vector Machine; LightGBM, Light Gradient Boosting Machine; DT, Decision Tree; KNN, K-Nearest Neighbors.

**Table S3.** **Predictive Discriminative Capacity Across ML Algorithms in the Training and Validation Cohorts**

| **Models** | **Training set** | | | | | | | |
| --- | --- | --- | --- | --- | --- | --- | --- | --- |
|  | **LR** | **XGBoost** | **LightGBM** | **RF** | **DT** | **MLP** | **SVM** | **KNN** |
| AUC  (95%CI) | 0.833 (0.801-0.865) | 0.860 (0.830-0.889) | 0.710 (0.669-0.750) | 0.877 (0.850-0.904) | 0.703 (0.663-0.743) | 0.676 (0.633-0.719) | 0.792  (0.746-0.838) | 0.872 (0.847-0.897) |
| Cutoff value | 0.097 | 0.15 | 0.138 | 0.131 | 0.372 | 0.411 | 0.109 | 0.165 |
| Accuracy  (95%CI) | 0.700  (0.685-0.715) | 0.743  (0.728-0.758) | 0.800  (0.777-0.822) | 0.759  (0.744-0.774) | 0.807  (0.787-0.826) | 0.727  (0.690-0.764) | 0.851  (0.844-0.859) | 0.737  (0.698-0.776) |
| Precision  (95%CI) | 0.292  (0.283-0.302) | 0.329  (0.317-0.341) | 0.366  (0.343-0.388) | 0.347  (0.332-0.361) | 0.372  (0.352-0.393) | 0.267  (0.240-0.294) | 0.470  (0.452-0.488) | 0.334  (0.300-0.368) |
| Recall  (95%CI) | 0.833  (0.814-0.853) | 0.833  (0.814-0.852) | 0.578  (0.523-0.632) | 0.849  (0.831-0.868) | 0.560  (0.512-0.608) | 0.585  (0.421-0.750) | 0.635  (0.619-0.651) | 0.835  (0.781-0.889) |
| F1 score  (95%CI) | 0.432  (0.425-0.440) | 0.471  (0.461-0.481) | 0.442  (0.437-0.447) | 0.492  (0.480-0.504) | 0.443  (0.438-0.448) | 0.343  (0.268-0.418) | 0.540  (0.529-0.550) | 0.471  (0.447-0.495) |

| **Models** | **Validation set** | | | | | | | |
| --- | --- | --- | --- | --- | --- | --- | --- | --- |
|  | **LR** | **XGBoost** | **LightGBM** | **RF** | **DT** | **MLP** | **SVM** | **KNN** |
| AUC  (95%CI) | 0.833  (0.736-0.929) | 0.818  (0.713-0.924) | 0.696 (0.571-0.821) | 0.835  (0.738-0.932) | 0.692  (0.569-0.816) | 0.679  (0.553-0.806) | 0.727  (0.566-0.887) | 0.813  (0.707-0.918) |
| Cutoff value | 0.097 | 0.15 | 0.138 | 0.131 | 0.372 | 0.411 | 0.109 | 0.165 |
| Accuracy  (95%CI) | 0.692  (0.660-0.724) | 0.721  (0.684-0.758) | 0.798  (0.769-0.827) | 0.729  (0.705-0.753) | 0.807  (0.779-0.834) | 0.730  (0.691-0.770) | 0.798  (0.771-0.825) | 0.708  (0.664-0.753) |
| Precision  (95%CI) | 0.285  (0.265-0.305) | 0.306  (0.286-0.326) | 0.364  (0.317-0.411) | 0.305  (0.290-0.320) | 0.375  (0.330-0.420) | 0.247  (0.181-0.313) | 0.365  (0.317-0.413) | 0.294  (0.268-0.320) |
| Recall  (95%CI) | 0.810  (0.732-0.888) | 0.770  (0.684-0.855) | 0.547  (0.492-0.601) | 0.753  (0.654-0.853) | 0.535  (0.485-0.585) | 0.583  (0.399-0.766) | 0.574  (0.513-0.636) | 0.751  (0.658-0.844) |
| F1 score  (95%CI) | 0.419  (0.395-0.444) | 0.431  (0.417-0.445) | 0.429  (0.397-0.461) | 0.429  (0.407-0.450) | 0.435  (0.402-0.467) | 0.322  (0.301-0.343) | 0.440  (0.397-0.483) | 0.415  (0.388-0.443) |

LR, Logistic Regression; RF, Random Forest; XGBoost, Extreme Gradient Boosting; MLP, Multilayer Perceptron; SVM, Support Vector Machine; LightGBM, Light Gradient Boosting Machine; DT, Decision Tree; KNN, K-Nearest Neighbors.

**Table S4.** Multivariate binary Logistic regression for HT

| **Variables** | **β** | **S.E** | **Z** | ***P*** | **OR (95%CI)** |
| --- | --- | --- | --- | --- | --- |
| Atrial fibrillation | 1.005 | 0.222 | 4.518 | <0.001 | 2.732 (1.767-4.226) |
| Blood glucose | 0.171 | 0.031 | 5.581 | <0.001 | 1.186 (1.117-1.259) |
| NLR | 0.075 | 0.020 | 3.832 | <0.001 | 1.078 (1.037-1.120) |
| ASPECTS | -0.503 | 0.089 | -5.632 | <0.001 | 0.604 (0.507-0.720) |
| NIHSS | 0.075 | 0.011 | 6.943 | <0.001 | 1.078 (1.055-1.101) |

OR: Odds Ratio; CI: Confidence Interval.

**Table S5.** Univariate Analysis of Key Variables for HT

| **Variables** | **AUC**  **(95%CI)** | **Accuracy (95%CI)** | **Sensitivity**  **(95%CI)** | **Specificity**  **(95%CI)** | **Cut off** | ***P* value** |
| --- | --- | --- | --- | --- | --- | --- |
| NLR | 0.696  (0.660-0.735) | 0.633  (0.555-0.729) | 0.753  (0.570-0.818) | 0.562  (0.518-0.750) | 3.063 | <0.001 |
| Atrial fibrillation | 0.599  (0.564-0.629) | 0.813  (0.794-0.836) | 0.305  (0.238-0.366) | 0.893  (0.878-0.911) | 1.000 | <0.001 |
| Blood glucose | 0.698  (0.648-0.734) | 0.652  (0.529-0.732) | 0.718  (0.554-0.816) | 0.609  (0.489-0.757) | 5.950 | <0.001 |
| ASPECTS | 0.733  (0.696-0.766) | 0.612  (0.585-0.643) | 0.581  (0.550-0.615) | 0.805  (0.756-0.859) | 8.000 | <0.001 |
| NIHSS | 0.767  (0.724-0.798) | 0.736  (0.664-0.782) | 0.695  (0.635-0.814) | 0.759  (0.649-0.797) | 10.000 | <0.001 |

AUC, area under the curve; CI, confidence interval.
